# Preparation, Antibacterial Potential, and Antibacterial Components of Fermented Compound Chinese Medicine Feed Additives

**DOI:** 10.3389/fvets.2022.808846

**Published:** 2022-03-24

**Authors:** Wanjie Zou, Honglan Huang, Huadong Wu, Yuandong Cao, Wei Lu, Yuyong He

**Affiliations:** ^1^Jiangxi Province Key Laboratory of Animal Nutrition/Engineering Research Center of Feed Development, Jiangxi Agricultural University, Nanchang, China; ^2^Forest Institution, Jiangxi Environmental Engineering Vocational College, Ganzhou, China; ^3^College of Animal Science and Technology, Jiangxi Agricultural University, Nanchang, China; ^4^Department of Technology, Jiangxi Jiabo Bioengineering Co. Ltd., Jiujiang, China

**Keywords:** Chinese medicine feed additive, fermentation, *in vitro* antibacterial activity, UHPLC-QE-MS based untargeted metabolomics, metabolic network

## Abstract

This experiment was conducted to compare the antibacterial ability and to identify the antibacterial components of different fermented compound Chinese medicine feed additives in order to develop one fermented compound Chinese medicine feed additive product that can effectively alleviate metritis, vaginitis, and mastitis of sows. The Oxford cup method and double dilution method were used to compare the antibacterial ability of three fermented compound Chinese medicine feed additives (A, B, and C). UHPLC-QE-MS-based untargeted metabolomics was used to identify the antibacterial components of fermented compound Chinese medicine feed additives. Results showed that among fermented compound Chinese medicine feed additives A, B, and C, additive A had the strongest ability to inhibit the growth of *Staphylococcus aureus, Salmonella cholerae suis, Escherichia coli*, and *Streptococcus agalactiae*. The MIC and MBC of additive A were the lowest for *Staphylococcus aureus* compared to that for the other three pathogens. The concentrations of 23 Chinese medicine ingredients (ellagic acid, guanine, camphor, L-valine, sinapine, dipropylphthalate, 3-hydroxy-5-isopropylidene-3,8-dimethyl-2,3,3a,4,5,8a-hexahydro-6(1H)-azulenone, 7-dihydroxy-2-(4-hydroxyphenyl)-8-[3,4,5-trihydroxy-6-(hydroxymethyl)oxan-2-yl]-6-(3,4,5-trihydroxyoxan-2-yl)chromen-4-one, acetylcholine, farrerol, pyrogallol, ethyl gallate, demethylwedelolactone, methyl gallate, kaempferide, gallic acid, eriodictyol, threonic acid, inositol, 3′,4′,7-trihydroxyflavanone, taxifolin, asiatic acid, and isorhamnetin) in additive A were significantly (*p* < 0.05 or *p* < 0.01) higher than those in additive B, respectively. It is concluded that the mixture composed of 23 active components in fermented compound Chinese medicine feed additive A plays an important role in inhibiting the growth of *Staphylococcus aureus, Salmonella cholerae suis, Escherichia coli*, and *Streptococcus agalactiae*.

## Introduction

Metritis, vaginitis, and mastitis of sows can cause a decrease in the number of pigs weaned per sow per year (PSY), because an unstable implantation of fertilized eggs developed by the chronic uteritis and vaginitis of sows will increase embryo death, block fetal intrauterine development, or bring conception into failure (1). Milk produced by sows with mastitis will decrease the survival rate and growth rate of suckling piglets by the increased diarrhea of suckling piglets (2). In the practice of treating metritis, vaginitis, and mastitis of sows over the past decades, in-feed antibiotics and intramuscular injection of antibiotics are often used (3, 4), but since July 1, 2020, the addition of antibiotics to feed is banned in China. Therefore, how to develop Chinese medicine feed additive products instead of antibiotics to control the bacterial pathogens of sows and improve sow's immunity is of great significance for the improvement of PSY. Previous studies indicated that metritis, vaginitis, and mastitis of sows and diarrhea of suckling piglets are often caused by the pathogens of *Escherichia coli, Staphylococcus aureus, Salmonella cholerae suis*, and *Streptococcus agalactiae* (5, 6), and these pathogens can be killed by many additives including nanozymes, organic acids, probiotics, herbs, and herb extracts under a certain concentration (7–11). Supplementation of Chinese medicine additives or Chinese medicine extracts to sows has effects in eliminating or alleviating metritis, vaginitis, and mastitis of sows and reducing diarrhea of suckling piglets (12–14), but the palatability of some untreated Chinese medicine additives or Chinese medicine extracts is not favorable owing to their bitter taste and feeding these additives or extracts directly to animals will decrease their feed intake. In addition, a large amount of residues will be produced and discarded during the production of Chinese medicine extracts, and the discarded residues will pollute the environment (15). The solid-state fermentation of compound Chinese medicine instead of extraction has many advantages including the better palatability of Chinese medicine additives and the less environmental pollution of Chinese medicine residues. The purpose of this study is to develop a fermented compound Chinese medicine feed additive formula by the *in vitro* antibacterial experiment and the identification of differential metabolites.

## Materials and Methods

### Formula of Compound Chinese Medicine Feed Additives

According to the components and pharmacological characteristics of traditional Chinese medicines, the following traditional Chinese medicines were selected to compose three compound Chinese medicine feed additives. Compound Chinese medicine feed additive A comprised 20 parts of *Houttuynia cordata*, 23 parts of *Folium artemisiae argyi*, 40 parts of *Thymifoious euphorbia* herb, 8 parts of *Cowherb* seed, 25 parts of *Potentilla discolor*, 45 parts of *Acalypha wilkesiana*, 10 parts of sugar, 26 parts of wheat, 15 parts of soybean meal, 0.03 parts of *Aspergillus niger*, and 1.5 parts of yeast. Compound Chinese medicine feed additive B comprised 20 parts of *Houttuynia cordata*, 15 parts of *Folium artemisiae argyi*, 40 parts of *Thymifoious euphorbia herb*, 25 parts of *Potentilla discolor*, 45 parts of *Motherwort*, 14 parts of *Licorice*, 18 parts of sugar, 8 parts of wheat, 8 parts of soybean meal, 0.05 parts of *Aspergillus niger*, and 1.0 parts of yeast. Compound Chinese medicine feed additive C comprised 25 parts of *Folium artemisiae argyi*, 12 parts of *Cowherb seed*, 30 parts of *Potentilla discolor*, 25 parts of *Thymifoious euphorbia herb*, 30 parts of *Acalypha wilkesiana*, 25 parts of sugar, 10 parts of soybean meal, 0.02 parts of *Aspergillus niger*, and 0.7 parts of yeast. Traditional Chinese medicines, wheat and soybean meal were ground into powder using a pulverizer to pass through 80-mesh sieves.

### Solid-State Fermentation of Compound Chinese Medicine Feed Additives

Tap water was firstly weighted at a ratio of 1:1 according to the weight of compound Chinese medicine feed additive and heated to 40°C to dissolve sugar, then *Aspergillus niger* and yeast were activated in this sugar solution for 30 min to produce a kind of fermentation liquid. The compound Chinese medicine feed additive was mixed with fermentation liquid, packed with plastic bags, and put into an oven for fermentation at 33°C for 36 h. The fermented additives were taken out and packed by vacuum sealing and stored at room temperature for 15 days.

### Preparation of Concentrated Liquid of Fermented Compound Traditional Chinese Medicine Feed Additive

Fifty gram of each fermented compound traditional Chinese medicine feed additive was soaked with 500 ml distilled water in a beaker for 1 h; then, the liquid mixture in the beaker was heated to boil and kept boiling for 1 h and filtered with 8 layers of gauze. The filter liquor was transferred into a glass cup, and the filter residue was washed into the beaker and boiled with 300 ml distilled water for 1 h; later, the mixture was filtered again. The twice-filtered liquors were mixed together and concentrated to 25 ml in an oven at 65°C with a final concentration of 2 g/ml; the concentrated liquor was sterilized by high-pressure steam and stored at −20°C for subsequent tests.

### Preparation of Bacterial Solution

*Streptococcus agalactiae* (GDMCC 1.768), *Escherichia coli* (GDMCC 1.176), *Staphylococcus aureus* (GDMCC 1.174), and *Salmonella cholerae suis* (GDMCC 1.163) were purchased from Guangdong Microbial Culture Collection Center. 0.3 ml brain heart infusion (BHI) broth was pipetted into the lyophilized tube containing *Streptococcus agalactiae*, then the tube was vortexed gently until the strain powder was completely dissolved; the suspension was pipetted into 5 ml BHI broth and cultured in an incubator at 37°C for 48 h. 0.3 ml Lennox broth (LB) was pipetted into the lyophilized tubes containing *Escherichia coli, Staphylococcus aureus*, and *Salmonella cholerae suis*; respectively, all tubes were vortexed gently until the strain powder was completely dissolved, then the suspension was respectively pipetted into 5 ml LB broth and cultured in an incubator at 37°C for 24 h. The optical density (OD) value of bacterial solution was measured at 660 nm to reach a final concentration of 1.5 × 10^8^ CFU/ml.

### *In vitro* Antibacterial Test

The tryptone soybean agar (TSA) was poured into the petri dishes; after agar solidification, a total of 0.1 μl *Escherichia coli* suspension (*Salmonella cholerae suis* or *Staphylococcus aureus* suspension was also spread according to this method) was uniformly spread on the surface of agar and three Oxford cups (7.8 ^*^ 6 ^*^ 10 mm) marked A, B, and C were placed on the agar medium in each petri dish. Two-hundred microliter of concentrated liquor A, B, or C was added to the corresponding Oxford cup; each strain had 3 replicates. All petri dishes were incubated at 37°C for 18 h and then taken out for the measurement of diameter of bacteriostatic circles. The blood culture medium was prepared to measure the abilities of concentrated liquor A, B, and C against the growth of *Streptococcus agalactiae* with the above method. Among the three concentrated liquors, the one with the best bacteriostatic effects against *Streptococcus agalactiae, Escherichia coli, Staphylococcus aureus*, and *Salmonella cholerae suis* was selected to determine its minimum inhibitory concentration (MIC) and minimum bactericidal concentration (MBC).

### MIC and MBC Determination

The double dilution method was used to determine the MIC. The selected concentrated liquor was successively diluted with LB broth (for *Escherichia coli, Staphylococcus aureus*, and *Salmonella cholerae suis*) or with BHI broth (for *Streptococcus agalactiae*) to give concentrations ranging from 1 to 0.0078125 g/ml in 8 culture tubes, and the ninth tube was only added with 2 ml LB broth as the positive control. Fifty microliter of bacteria solution was added into each tube, and all tubes were incubated for 18 h at 37°C. The lowest concentration of the selected concentrated liquor that caused a visible inhibitory effect was defined as the MIC. The MBC was determined by incubating 10-μl samples from each tube with an inhibitory effect on plates with TSA (for *Escherichia coli, Staphylococcus aureus*, and *Salmonella cholerae suis*) or with blood culture medium (for *Streptococcus agalactiae*) at 37°C for 18 h, with the lowest concentration of sample with no visible sign of growth determined as the MBC. All experiments were conducted in three replicates.

### UHPLC-QE-MS-Based Untargeted Metabolomics

Metabolomics was applied using 1,290 ultra-high-performance liquid chromatography (Agilent, CA, USA) coupled with Q Exactive focus MS/MS (Thermo Fisher Scientific, Waltham, MA, USA). Chromatographic separations were performed using a Waters ACQUITY system equipped with an ACQUITY UPLC BEH C18 column (1.7 μm, 2.1 × 100 mm). The mobile phase consisted of 0.1% formic acid water (A) and 0.1% formic acid acetonitrile (B) at the flow rate of 0.5 ml/min, and the injection volume was 4 ml. The gradient program was as follows: 85–25% B (0–11.0 min), 25–2% B (11.0–12.0 min), 2–2% B (12.0–14.0 min), 2–85% B (14.0–14.1 min), 85–85% B (14.1–15.0 min), and 85–85% B (15.0–16.0 min). The ESI source was applied to analyze the chemical composition in both positive and negative ion modes with full scan/ddMS2. The MS parameters were set as follows: the scan range was 50–1,000 m/z, the spray voltages were set at 4.0 and 3.6 kV in positive and negative modes, respectively, sheath gas was 35 arb, auxiliary gas was 10 arb, the capillary temperature was 400°C, the maximum injection time for MS1 and ddMS2 was 100 and 45 ms, respectively, and the resolutions for MS1 and ddMS2 were 70,000 and 17,500, respectively; putative molecules of interest were fragmented using three different collision energies (10, 20, and 40 eV).

### Multivariate Statistical Analysis and Metabolite Identification

Raw data of UHPLC-QE-MS were processed using the XCMS online platform, and the qualified data were uploaded to SIMCA-P (Version 16.0.2, Sartorius Stedim Data Analytics AB, Umea, Sweden) for multivariate statistical analysis. Partial least-square-discriminate analysis (PLS-DA) and orthogonal partial least-square-discriminate analysis (OPLS-DA) were performed to find out the metabolic distinction. The variable importance of projection (VIP) values was used to characterize the contributions of the metabolites to the change rates of Y variety in OPLS-DA, and metabolites that meet these criteria including MS2 score > 0.80, VIP > 1.0, and *p* < 0.05 were selected as the differential variables; metabolite identification was performed by searching the in-house database and web databases (METLIN, HMDB, PubChem, and ChemSpider).

### Network Analysis of Targeted Differentially Metabolites

To further investigate the mechanism of targeted differential metabolites, the network pharmacology approach was introduced to uncover the related pathways, compounds, enzymes, and reactions. Compounds with MS2 score > 0.80, VIP > 1.0, fold change > 1, and o <0.05 were selected as the target differential metabolites and used to construct the network with FELLA R package (16). The nodes in the network were compounds, enzymes, pathways, modules, and reactions, and the relationship between them was represented by the lines between the nodes.

### Statistical Analysis

Statistical analysis was performed by SPSS 17.0 (version 17.0, USA), comparisons between two groups were made with Student's *t*-test; the value at *p* < 0.05 was considered as significance threshold, and the results were presented as mean ± SE.

## Results

### *In vitro* Bacteriostatic Effect of Different Fermented Compound Chinese Medicine Feed Additives

**Figure 1:**
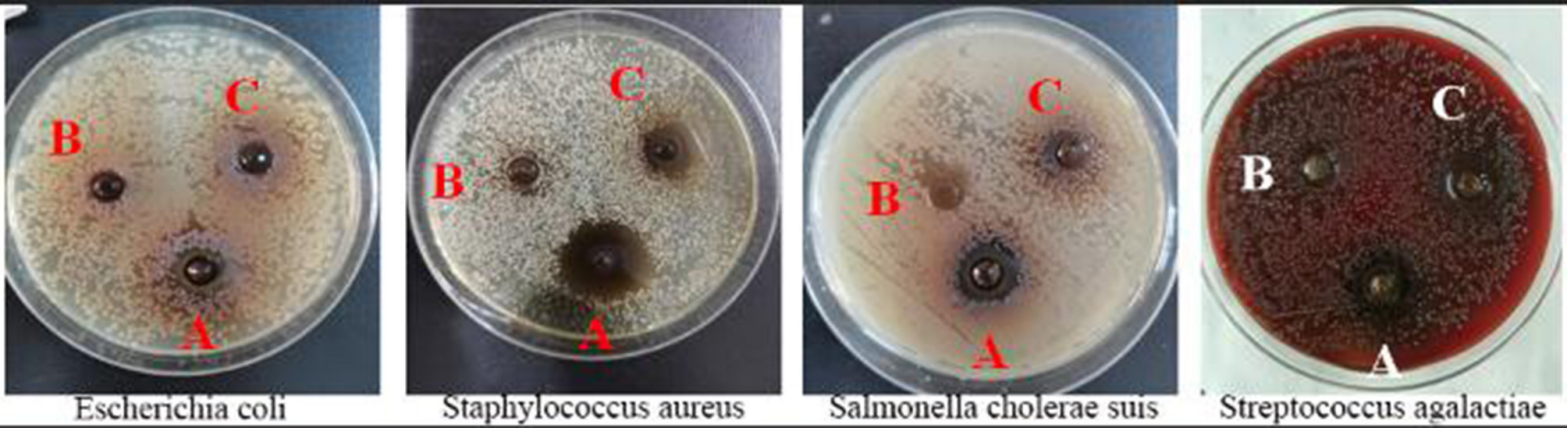
Diagram of the bacteriostatic circle of three fermented compound Chinese medicine feed additives.

The abilities of additives A, B, and C in inhibiting the growth of *Streptococcus agalactiae, Escherichia coli, Staphylococcus aureus*, and *Salmonella cholerae suis* were different and ranked as follows: A > C > B. Results in Figure 1 and Table 1 indicated that additive A was the most effective in inhibiting the growth of *Staphylococcus aureus* but had the worst effect in preventing the growth of *Streptococcus agalactiae*, additive B had no bacteriostatic effect, and additive C had the ability in controlling the growth of *Streptococcus agalactiae, Staphylococcus aureus*, and *Salmonella cholerae suis* but had no bacteriostatic effect on *Escherichia coli*.

**Table 1:** *In vitro* antibacterial effects of different fermented compound Chinese medicine feed additives.

**Concentrated liquors**	**Bacteriostatic circle diameter (mm)**			
	***Escherichia coli***	***Staphylococcus aureus***	***Salmonella cholerae suis***	***Streptococcus agalactiae***
A	11.41 ± 0.27^A^	21.54 ± 0.27^A^	14.66 ± 0.48^A^	8.83 ± 0.24^A^
B	0.00 ± 0.00^B^	0.00 ± 0.00^C^	0.00 ± 0.00^C^	0.00 ± 0.00^C^
C	0.00 ± 0.00^B^	11.92 ± 0.10^B^	7.92 ± 0.41^B^	7.88 ± 0.10^B^

*In the same column, values with the same or no letter superscripts mean no significant difference (p > 0.05); values with different capital letter superscripts mean significant difference (p <0.01)*.

### MIC and MBC of Fermented Compound Chinese Medicine Feed Additive A

Fermented compound Chinese medicine feed additive A was used to determine MIC and MBC because additive A had the best bacteriostatic effect compared to the other two additives, and data in Table 2 showed that additive A had the lowest MIC and MBC for *Staphylococcus aureus* among all tested bacteria.

**Table 2:** MIC and MBC of fermented compound Chinese medicine feed additive A, g/ml.

**Concentrated liquor**	***Escherichia coli***		***Salmonella cholerae suis***		***Staphylococcus aureus***		***Streptococcus agalactiae***	
	**MIC**	**MBC**	**MIC**	**MBC**	**MIC**	**MBC**	**MIC**	**MBC**
A	0.1250	0.2500	0.1250	0.2500	0.0625	0.1250	0.1250	0.2500

### Major Antibacterial Components of Fermented Compound Chinese Medicine Feed Additive A

In order to find out why additive A had better antibacterial ability than additive B, the metabolomics untargeted analysis was performed to identify the differential metabolites between additives A and B, and metabolites with MS2 score > 0.80, VIP > 1.0, fold change > 1, and *p* < 0.05 were selected as the target compounds. Results in Table 3 indicated that additive A had significantly higher concentrations of ellagic acid, guanine, camphor, L-valine, sinapine, dipropylphthalate, 3-hydroxy-5-isopropylidene-3,8-dimethyl-2,3,3a,4,5,8a-hexahydro-6(1H)-azulenone, 7-dihydroxy-2-(4-hydroxyphenyl)-8-[3,4,5-trihydroxy-6-(hydroxymethyl)oxan-2-yl]-6-(3,4,5-trihydroxyoxan-2-yl)chromen-4-one, acetylcholine, farrerol, pyrogallol, ethyl gallate, demethylwedelolactone, methyl gallate, kaempferide, gallic acid, eriodictyol, threonic acid, inositol, 3′,4′,7-trihydroxyflavanone, taxifolin, asiatic acid, and isorhamnetin than additive B.

**Table 3:** Information of differential metabolites with fold change (A/B) large than 1.

**MS 2 name**	**MS2 score**	**Compound A**	**Compound B**	**Fold change (A/B)**	**VIP**	***p*-value**
**Positive ion model**						
Ellagic acid	1.00	89970.65 ± 2047.27	6.03 ± 0.49	14920.51	1.44	0.048
Guanine	1.00	893248.98 ± 12338.31	630877.16 ± 30346.84	1.42	1.41	0.001
Camphor	0.97	165777.89 ± 17351.72	95213.63 ± 7517.58	1.74	1.30	0.020
L-Valine	0.96	8283283.61 ± 991023.05	4722270.50 ± 194648.71	1.75	1.30	0.024
Sinapine	0.94	84351.79 ± 15192.09	25909.49 ± 4472.36	3.26	1.33	0.021
Dipropylphthalate	0.94	16261.57 ± 3902.65	3521.70 ± 1481.38	4.62	1.21	0.038
3-Hydroxy-5-isopropylidene-3,8-dimethyl-2,3,3a,4,5,8a-hexahydro-6(1H)-azulenone	0.92	14268.67 ± 1464.43	956.23 ± 86.74	14.92	1.43	0.012
5,7-Dihydroxy-2-(4-hydroxyphenyl)-8-[3,4,5-trihydroxy-6-(hydroxymethyl)oxan-2-yl]-6-(3,4,5-trihydroxyoxan-2-yl)chromen-4-one	0.92	95382.59 ± 7467.40	57754.10 ± 484.49	1.65	1.38	0.037
Acetylcholine	0.91	4467186.16 ± 430688.27	47437.4 ± 4997.85	94.17	1.44	0.009
Farrerol	0.81	91502.75 ± 10528.55	41296.36 ± 4047.05	2.22	1.37	0.011
**Negative ion model**						
Pyrogallol	1.00	217175.34 ± 26949.40	85161.91 ± 7780.19	2.55	1.12	0.047
Ethyl gallate	0.99	15482400.23 ± 2865182.97	49027.25 ± 4807.40	315.79	1.42	0.033
Demethylwedelolactone	0.98	178662.97 ± 15761.08	55273.17 ± 12557.93	3.23	1.32	0.004
Methyl gallate	0.98	334131.77 ± 26014.26	3559.89 ± 977.21	93.86	1.21	0.006
Kaempferide	0.98	386085.28 ± 48610.66	238295.59 ± 10102.16	1.62	1.24	0.041
Gallic acid	0.98	17732911.31 ± 246955.79	63766.36 ± 4747.67	278.09	1.42	0.000
Eriodictyol	0.92	408383.73 ± 16484.25	220608.55 ± 11549.24	1.85	1.39	0.001
Threonic acid	0.92	1163675.16 ± 61626.38	539744.37 ± 78673.13	2.16	1.32	0.003
Inositol	0.86	1927229.94 ± 103370.35	1419620.73 ± 40982.28	1.36	1.33	0.010
3′,4′,7-Trihydroxyflavanone	0.85	5546.82 ± 981.38	2073.68 ± 610.07	2.67	1.21	0.040
Taxifolin	0.84	73540.26 ± 12851.85	15961.30 ± 2362.12	4.61	1.36	0.012
Asiatic acid	0.84	25611.49 ± 3923.63	1894.61 ± 459.78	13.52	1.38	0.025
Isorhamnetin	0.81	204543.52 ± 25406.85	46807.20 ± 2097.82	4.37	1.40	0.024

### Metabolic Network Analyses

**Figure 2:**
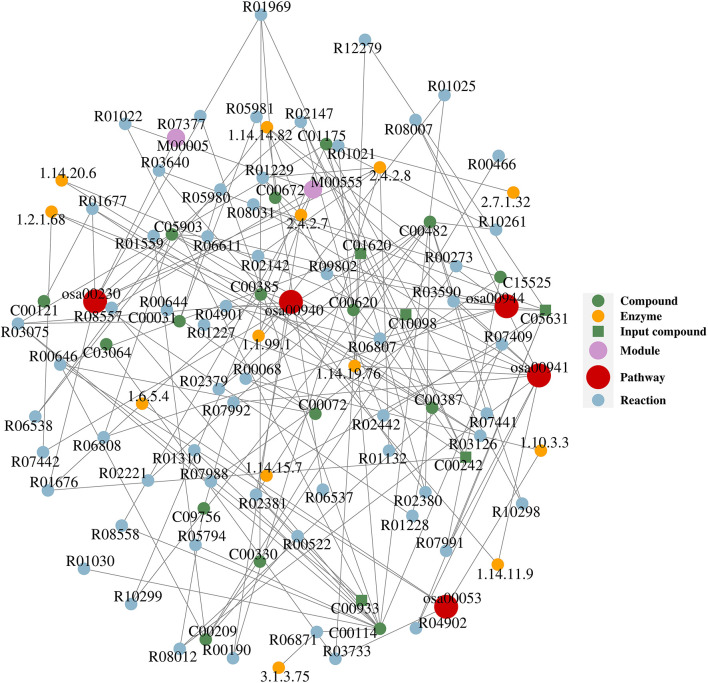
The compound–reaction–enzyme–pathway networks of the targeted metabolites. Compounds: C00031 (D-glucose), C00072 (ascorbate), C00114 (choline), C00121 (D-ribose), C00209 (oxalate), C00242 (guanine), C00330 (deoxyguanosine), C00385 (xanthine), C00387 (guanosine), C00482 (sinapate), C00620 (alpha-D-ribose 1-phosphate), C00672 (2-deoxy-D-ribose 1-phosphate), C00933 (sinapine), C01175 (1-O-sinapoyl-beta-D-glucose), C01620 (threonate), C03064 (3-dehydro-L-threonate), C05631 (eriodictyol), C05903 (kaempferol), C09756 (homoeriodictyol), C10098 (kaempferide), C15525 (eriodictyol chalcone). Reactions: R00068 (L-ascorbate:oxygen oxidoreductase), R00190 (AMP:diphosphate phospho-D-ribosyltransferase), R00273 (oxalate:oxygen oxidoreductase), R00466 (glyoxylate:oxygen oxidoreductase), R00522 (oxalate carboxy-lyase), R00644 (L-ascorbate:hydrogen-peroxide oxidoreductase), R00646 (ascorbate + oxygen + H_2_O <=> threonate + oxalate), R01021 (ATP:choline phosphotransferase), R01022 (choline:oxygen 1-oxidoreductase), R01025 (choline:acceptor 1-oxidoreductase), R01132 (IMP:diphosphate phospho-D-ribosyltransferase), R01227 (guanosine 5′-monophosphate phosphohydrolase), R01228 (ATP:guanosine 5′-phosphotransferase), R01229 (GMP:diphosphate 5-phospho-alpha-D-ribosyltransferase), R01310 (phosphatidylcholine phosphatidohydrolase), R01559 (succinyl-CoA:oxalate CoA-transferase), R01676 (guanine aminohydrolase), R01677 (guanosine ribohydrolase), R01969 (deoxyguanosine:orthophosphate ribosyltransferase), R02142 (XMP:pyrophosphate phosphoribosyltransferase), R02147 (guanosine:phosphate alpha-D-ribosyltransferase), R02221 [sinapate:CoA ligase (AMP-forming)], R02379 (S-adenosyl-L-methionine:3,4-dihydroxy-trans-caffeoyl-coenzyme), R02380 (UDPglucose:sinapate D-glucosyltransferase), R02381 (sinapoylcholine sinapohydrolase), R02442 (naringenin,[reduced NADPH—hemoprotein reductase), R03075 (1-O-(4-hydroxy-3,5-dimethoxycinnamoyl)-beta-dimethoxycinnamoyl), R03126 (dihydroflavonol,2-oxoglutarate:oxygen oxidoreductase), R03590 (flavanone,2-oxoglutarate:oxygen oxidoreductase), R03640 (flavanone,2-oxoglutarate:oxygen oxidoreductase), R03733 (L-threonate:NAD+ 3-oxidoreductase), R04901 ((2S)-flavan-4-ol:NADP+ 4-oxidoreductase), R04902 (eriodictyol,[reduced NADPH—hemoprotein reductase), R05794 (CDP-diacylglycerol:choline O-phosphatidyltransferase), R05980 (Glc2Man9GlcNAc2-[protein] 3-alpha-glucohydrolase), R05981 (GlcMan9GlcNAc2-[protein] 3-alpha-glucohydrolase), R06537 (apigenin,[reduced NADPH—hemoprotein reductase), R06538 (kaempferol,[reduced NADPH—hemoprotein reductase), R06611 (UDPglucose:flavonol 3-O-D-glucosyltransferase), R06807 (S-adenosyl-L-methionine:kaempferol 4'-O-methyltransferase), R06808 (UDP-alpha-D-galactose:kaempferol 3-O-beta-D-galactose), R06871 (phosphocholine phosphohydrolase), R07377 (phosphatidylcholine + L-Serine <=> phosphatidylcholine), R07409 (choline,reduced-ferredoxin:oxygen oxidoreductase), R07441 (sinapoyl aldehyde:NAD oxidoreductase), R07442 (sinapoyl aldehyde:NAD oxidoreductase), R07988 (malonyl-CoA:caffeoyl-CoA malonyltransferase), R07991 (eriodictyol chalcone <=> eriodictyol), R07992 (homoeriodictyol chalcone <=> homoeriodictyol), R08007 (UDP-alpha-D-glucose:2′,3,4,4′,6′-pentahydroxy heptanal), R08012 (eriodictyol <=> homoeriodictyol), R08031 (eriodictyol chalcone <=> homoeriodictyol chalcone), R08557 (choline + NAD+ <=> betaine aldehyde + NADH), R08558 (choline + NADP+ <=> betaine aldehyde + NADPH), R09802 (UDP-L-rhamnose:kaempferol 3-O-rhamnosyltransfase), R10261 (1-O-sinapoyl-beta-D-glucose:cyanidin-3-O-beta- D-glucoside), R10298 (1-O-sinapoyl-beta-D-glucose:pelargonidin-3-O- malonylglucoside), R10299 (1-O-sinapoyl-beta-D-glucose:delphinidin-3-O- beta -D-glucoside), R12279 (eriodictyol,[reduced NADPH—hemoprotein reductase). Enzymes: 1.1.99.1 (choline dehydrogenase), 1.10.3.3 (L-ascorbate oxidase), 1.14.11.9 (flavanone 3-dioxygenase), 1.14.14.82 (flavonoid 3′-monooxygenase), 1.14.15.7 (choline monooxygenase), 1.14.19.76 (flavone synthase II), 1.14.20.6 (flavonol synthase), 1.2.1.68 (coniferyl-aldehyde dehydrogenase), 1.6.5.4 [monodehydroascorbate reductase (NADH)], 2.4.2.7 (adenine phosphoribosyltransferase), 2.4.2.8 (hypoxanthine phosphoribosyltransferase), 2.7.1.32 (choline kinase), 3.1.3.75 (phosphoethanolamine/phosphocholine phosphatase). Pathways: osa00053 (ascorbate and aldarate metabolism), osa00230 (purine metabolism), osa00940 (phenylpropanoid biosynthesis), osa00941 (flavonoid biosynthesis), osa00944 (flavone and flavonol biosynthesis).

Network analysis was conducted in order to reveal the molecular network and mechanism of the interaction of the KEGG pathways with related compounds, enzymes, modules, and reactions. Twenty-three differential compounds were mapped with the KEGG database, and only five compounds (guanine-C00242, eriodictyol-C05631, kaempferide-C10098, threonic acid-C01620, and sinapine-C00933) indicated as green square in Figure 2 were successfully mapped onto 5 KEGG metabolic pathways (purine metabolism-osa00230, flavonoid biosynthesis-osa00941, flavone and flavonol biosynthesis-osa00944, ascorbate and aldarate metabolism-osa00053, phenylpropanoid biosynthesis-osa00940). Figure 2 shows that guanine, eriodictyol, kaempferide, threonic acid, and sinapine can be converted to the following compounds *via* the related enzymes and reactions: D-glucose, ascorbate, choline, D-ribose, oxalate, deoxyguanosine, xanthine, guanosine, sinapate, alpha-D-ribose 1-phosphate, 2-deoxy-D-ribose 1-phosphate, 1-O-sinapoyl-beta-D-glucose, 3-dehydro-L-threonate, kaempferol, homoeriodictyol, and eriodictyol chalcone.

## Discussion

Previous studies indicated that compounds listed in Table 3 have bacteriostatic and/or bactericidal effects. Ellagic acid has strong inhibitory effects on *Staphylococcus aureus, Salmonella cholerae suis, Escherichia coli, Streptococcus*, and *Bacillus cereus* (17, 18). Purine and amino acids can inhibit the growth of bacteria in the form of riboswitches (a kind of RNA sequences that can bind to small molecular ligands and regulate gene expression, which can regulate the corresponding downstream gene expression after binding to purine and amino acids and other ligands), so as to achieve the antibacterial and bactericidal effects (19–21). Camphor can inhibit the growth of *Salmonella cholerae suis* and help animals to excrete toxins (22), while sinapine can inhibit the growth of *Escherichia coli* (23). 5,7-Dihydroxy-2-(4-hydroxyphenyl)-8-[3,4,5-trihydroxy-6-(hydroxymethyl)oxan-2-yl]-6-(3,4,5-trihydroxyoxan-2-yl)chromen-4-one named as isoxiafota glycoside has inhibitory effects on *Escherichia coli, Proteus vulgaris, Pseudomonas aeruginosa, Staphylococcus aureus*, and *Enterococcus faecalis* (24). Farrerol can inhibit the growth of *Staphylococcus aureus* (25); however, pyrogallol and demethylwedelolactone (norwedelide) not only can inhibit the growth of *Staphylococcus aureus* but also can prevent the growth of *Escherichia coli* and *Salmonella* (26, 27). Kaempferide, gallic acid, and taxifolin have good antibacterial effects on *Staphylococcus aureus, Escherichia coli, dysentery bacilli, Pseudomonas aeruginosa*, and *Streptococcus* (28–30). Ethyl gallate can inhibit the growth of *Staphylococcus aureus* and *Escherichia coli* (31, 32). Eriodictyol and inositol have good inhibitory effects on *Escherichia coli, Staphylococcus aureus*, and *Pseudomonas aeruginosa* (33, 34). Trihydroxyflavanone, asiatic acid, and isorhamnetin have good inhibitory or killing effects on *Shigella dysentery bacteria, Salmonella, Streptococcus, Staphylococcus aureus*, and *Escherichia coli* (35–37). These plant-derived active ingredients exert bacteriostatic and bactericidal effects through inhibiting the production of extracellular polymers (EPS), reducing bacterial adhesion, destroying the integrity and permeability of bacterial cell membrane, inactivating enzymes of bacteria, changing cell metabolism, blocking nucleic acid synthesis, and enhancing animal immunity (38, 39). Figure 2 shows that the differential metabolites which have been mapped onto metabolic pathways can be converted to D-glucose and 2-deoxy-D-ribose-1-phosphate, exposure to high concentration of glucose exerted bacteriostatic or bactericidal effects by destroying ribosome assembly of bacteria (40), and 2-deoxy-d-ribose-1-phosphate can achieve the purpose of bacteriostasis by inhibiting the synthesis of bacterial nucleic acid (41).

Results of this experiment showed that fermented compound Chinese medicine feed additive A had significantly higher concentrations of ellagic acid, guanine, camphor, L-valine, sinapine, dipropylphthalate, 3-hydroxy-5-isopropylidene-3,8-dimethyl-2,3,3a,4,5,8a-hexahydro-6(1H)-azulenone, 7-dihydroxy-2-(4-hydroxyp-henyl)-8-[3,4,5-trihydroxy-6-(hydroxymethyl)oxan-2-yl]-6-(3,4,5-trihydroxyoxan-2-yl)chromen-4-one, acetylcholine, farrerol, pyrogallol, ethyl gallate, demethylwedelolactone, methyl gallate, kaempferide, gallic acid, eriodictyol, threonic acid, inositol, 3′,4′,7-trihydroxyflavanone, taxifolin, asiatic acid, and isorhamnetin than fermented compound Chinese medicine feed additive B; most of them and their derivatives have strong bacteriostatic and/or bactericidal effects against pathogens, and this is the reason why fermented compound Chinese medicine feed additive A has better bacteriostatic and/or bactericidal effects than fermented compound Chinese medicine feed additive B.

## Conclusions

Additive A had the strongest ability to inhibit the growth of *Staphylococcus aureus, Salmonella cholerae suis, Escherichia coli*, and *Streptococcus agalactiae*; the mixture composed of 23 active components in fermented compound Chinese medicine feed additive A exerted bacteriostatic and/or bactericidal effects on the tested pathogens. The sensitivity of these four bacteria to fermented compound traditional Chinese medicine feed additive A is ranked as *Staphylococcus aureus* > *Salmonella cholerae suis* > *Escherichia coli* > *Streptococcus agalactiae*.

## Data Availability Statement

The original contributions presented in the study are included in the article/supplementary material, further inquiries can be directed to the corresponding authors.

## Author Contributions

YH and WL: conception, experimental design, and manuscript preparation. WZ, HH, and YC: investigation. WZ and HW: data analysis. All authors have read and agreed to the published version of the manuscript.

## Funding

This work was supported by the Key Research and Development Plan of Jiangxi Province (20192BBFL60021).

## Conflict of Interest

Jiangxi Jiabo Bioengineering Co., Ltd., was affiliated with this study. The authors declare that the research was conducted in the absence of any commercial or financial relationships that could be construed as a potential conflict of interest.

## Publisher's Note

All claims expressed in this article are solely those of the authors and do not necessarily represent those of their affiliated organizations, or those of the publisher, the editors and the reviewers. Any product that may be evaluated in this article, or claim that may be made by its manufacturer, is not guaranteed or endorsed by the publisher.

## References

[1] Mauch C Bilkei G. The influence of prepartal bacteriuria on the reproductive performance of the sow. Dtsch Tierarztl Wochenschr. (2004) 111:166–9. 15171603

[2] Kaiser M Jacobson M Andersen PH Bækbo P Cerón JJ Dahl J. Inflammatory markers before and after farrowing in healthy sows and in sows affected with postpartum dysgalactia syndrome. BMC Vet Res. (2018) 14:83. 10.1186/s12917-018-1382-7 29530043 PMC5848515

[3] Hirsch AC Philipp H Kleemann R. Investigation on the efficacy of meloxicam in sows with mastitis-metritis-agalactia syndrome. J Vet Pharmacol Ther. (2003) 26:355–60. 10.1046/j.1365-2885.2003.00524.x 14633188

[4] Alexopoulos C Fthenakis GC Burriel A Bourtzi-Hatzopoulou E Kritas SK Sbiraki A. The effects of the periodical use of in-feed chlortetracycline on the reproductive performance of gilts and sows of a commercial pig farm with a history of clinical and subclinical viral and bacterial infections. Reprod Domest Anim. (2003) 38:187–92. 10.1046/j.1439-0531.2003.00415.x 12753551

[5] Kemper N Bardehle D Lehmann J Gerjets I Looft H Preissler R. The role of bacterial pathogens in coliform mastitis in sows. Berl Munch Tierarztl Wochenschr. (2013) 126:130–6. 10.2376/0005-9366-126-130 23540195

[6] Rodríguez PYDLF Martin LOM Muñoz EC Imberechts H Butaye P Goddeeris BM. Several enteropathogens are circulating in suckling and newly weaned piglets suffering from diarrhea in the province of Villa Clara, Cuba. Trop Anim Health Prod. (2013) 45:435–40. 10.1007/s11250-012-0236-8 22843242 PMC7089418

[7] Shi SR Wu S Shen YR Zhang S Xiao YQ He X. Iron oxide nanozyme suppresses intracellular *Salmonella enteritidis* growth and alleviates infection in vivo. Theranostics. (2018) 8:6149–62. 10.7150/thno.29303 30613289 PMC6299686

[8] Xiao YQ Zhang S Tong HB Shi SR. Comprehensive evaluation of the role of soy and isoflavone supplementation in humans and animals over the past two decades. Phytother Res. (2018) 32:384–94. 10.1002/ptr.5966 29193539

[9] Zhang S Zhong G Shao D Wang Q Hu Y Wu TX. Dietary supplementation with Bacillus subtilis promotes growth performance of broilers by altering the dominant microbial community. Poultry Sci. (2021) 100:100935. 10.1016/j.psj.2020.12.032 33652528 PMC7936199

[10] Hu Y Wang LD Shao D Wang Q Wu YY Han YM. Selectived and reshaped early dominant microbial community in the cecum with similar proportions and better homogenization and species diversity due to organic acids as AGP alternatives mediate their effects on broilers growth. Front Microbiol. (2020) 10:2948. 10.3389/fmicb.2019.02948 31993028 PMC6971172

[11] Zhang S Shen YR Wu S Xiao YQ He Q Shi SR. The dietary combination of essential oils and organic acids reduces *Salmonella enteritidis* in challenged chicks. Poultry Sci. (2019) 98:6349–55. 10.3382/ps/pez457 31393588 PMC8913765

[12] Chang CH Chen YS Chiou MT Su CH Chen DS Tsai CE. Application of scutellariae radix, gardeniae fructus, and probiotics to prevent *salmonella enterica serovar choleraesuis* infection in swine. Evid Based Complement Alternat Med. (2013) 2013:568528. 10.1155/2013/568528 23533497 PMC3600312

[13] Shen X Niu XD Li G Deng XM Wang JF. Amentoflavone ameliorates *Streptococcus suis*-induced infection *in vitro* and *in vivo*. Appl Environ Microbiol. (2018) 84:e01804–18. 10.1128/AEM.01804-18 30315078 PMC6275344

[14] Gong JH Yin FG Hou YQ Yin Y. Review: Chinese herbs as alternatives to antibiotics in feed for swine and poultry production: potential and challenges in application. Can J Anim Sci. (2014) 94:223–41. 10.4141/cjas2013-144

[15] Guo QQ Li J Chen GY Guo X Cheng ZJ Yan BB. A comprehensive comparison study: the impacts of gasifying agents and parameters on Chinese herb medicine residue gasification. Waste Biomass Valori. (2021) 12:3059–73. 10.1007/s12649-020-01037-x

[16] Picart-Armada S Fernández-Albert F Vinaixa M Yanes O Perera-Lluna A. ELLA: an R package to enrich metabolomics data. BMC bioinformatics. (2018) 19:538. 10.1186/s12859-018-2487-5 30577788 PMC6303911

[17] Ghudhaib KK Hanna ER Jawad AH. Effect of ellagic acid on some types of pathogenic bacteria. Al-Nahrain J Sci. (2010) 13:79–85. 10.22401/JNUS.13.2.09

[18] Zhu HL Chen G Chen SN Wang QR Wan L Jian SP. Characterization of polyphenolic constituents from *Sanguisorba officinalis L*. and its antibacterial activity. Eur Food Res Technol. (2019) 245:1487–98. 10.1007/s00217-019-03276-2

[19] Batey RT. Structure and mechanism of purine-binding riboswitches. Q Rev Biophys. (2012) 45:345–81. 10.1017/S0033583512000078 22850604 PMC3760793

[20] Richards J Belasco JG. Riboswitch control of bacterial RNA stability. Mol microbial. (2021) 116:361–5. 10.1111/mmi.14723 33797153 PMC10367942

[21] Blount KF Breaker RR. Riboswitches as antibacterial drug targets. Nat Biotechnol. (2006) 24:1558–64. 10.1038/nbt1268 17160062

[22] Insawang S Pripdeevech P Tanapichatsakul C Khruengsai S Monggoot S Nakham T. Essential oil compositions and antibacterial and antioxidant activities of five *Lavandula stoechas* cultivars grown in Thailand. Chem biodivers. (2019) 16:e1900371. 10.1002/cbdv.201900371 31464066

[23] Adegbeye MJ Elghandour MMMY Faniyi TO Perez NR Barbabosa-Pilego A Zaragoza-Bastida A. Antimicrobial and antihelminthic impacts of black cumin, pawpaw and mustard seeds in livestock production and health. Agroforest Syst. (2020) 94:1255–68. 10.1007/s10457-018-0337-0

[24] Song CW Huang L Rong L Zhou ZW Peng XH Yu SG. Anti-hyperglycemic effect of Potentilla discolor decoction on obese-diabetic(ob-db) mice and its chemical composition. Fitoterapia. (2012) 83:1474–83. 10.1016/j.fitote.2012.08.013 22960384

[25] Yang ZT Fu YH Liu B Zhou ES Liu ZC Song XJ. Farrerol regulates antimicrobial peptide expression and reduces *staphylococcus aureus* internalization into bovine mammary epithelial cells. Microb Pathog. (2013) 65:1–6. 10.1016/j.micpath.2013.08.002 24036182

[26] Udayashankar AC Nandhini M Rajini SB Prakash HS. Pharmacological significance of medicinal herb Eclipta alba L-A review. Int J Pharm Sci Res. (2019) 10:3592–606. 10.13040/IJPSR.0975-8232.10(8).3592-06

[27] Khatua S Acharya K. Functional ingredients and medicinal prospects of ethanol extract from macrocybe lobayensis. Pharmacogn J. (2018) 10:1154–8. 10.5530/pj.2018.6.197

[28] Eloff JN Teffo LS Toms RB Aderogba AM. The possible interaction between an edible insect and five antibacterial kaempferol methyl ethers isolated from *Dodonaea viscosa* Jacq. var angustifolia (Sapindaceae) leaf extracts. Planta Med. (2007) 73:61–7. 10.1055/s-2007-986732

[29] Borges A Ferreira C Saavedra MJ Simões M. Antibacterial activity and mode of action of ferulic and gallic acids against pathogenic bacteria. Microb Drug Resist. (2013) 19:256–65. 10.1089/mdr.2012.0244 23480526

[30] Ahamad ST Lakshmi T Shanmugam R Roy A Gurunadhan D Geetha RV. Antibacterial activity of taxifolin isolated from acacia catechu leaf extract-an *in vitro* study. Indian J Public Health Res Dev. (2019) 10:3540. 10.5958/0976-5506.2019.04135.4

[31] Li K Lin Y Li B Pan TW Wang F Yuan RQ. Antibacterial constituents of Fructus Chebulae Immaturus and their mechanisms of action. BMC Complement Altern Med. (2016) 16:183. 10.1186/s12906-016-1162-5 27368700 PMC4930599

[32] Olmedo-Juárez A Briones-Robles TI Zaragoza-Bastida A Zamilpa A Ojeda-Ramírez D de Gives PM. Antibacterial activity of compounds isolated from *Caesalpinia coriaria* (Jacq) Willd against important bacteria in public health. Microb Pathog. (2019) 136:103660. 10.1016/j.micpath.2019.103660 31398533

[33] Ciric A Kariotli A Glamoclija J Sokovic M Skaltsa H. Antimicrobial activity of secondary metabolites isolated from Centaurea spruneri Boiss & Heldr. J Serb Chem Soc. (2011) 76:27–34. 10.2298/JSC100127008C

[34] Pudutha A Venkatesh K Chakrapani P Chandra Sekhar Singh B Kumar P Rani AR. Traditional uses, phytochemistry and pharmacology of an endangered plant-decalepis hamiltonii. Wight and Arn Int J Pharm Sci Rev Res. (2014) 24:268–78.

[35] Masoko P Picard J Howard RL Mampuru LJ Eloff JN. *In Vivo* antifungal effect of combretum and terminalia species extracts on cutaneous wound healing in immunosuppressed rats. Pharm Biol. (2010) 48:621–32. 10.3109/13880200903229080 20645734

[36] Liu WH Liu TC Mong MC. Antibacterial effects and action modes of asiatic acid. Biomedicine. (2015) 5:16. 10.7603/s40681-015-0016-7 26280399 PMC10723755

[37] Abdi B Getaneh E Assefa T Dekebo A Tesso H Abdo T. Chemical constituents of the roots extract of dryopteris schimperiana and evaluation for antibacterial and radical scavenging activities. Ethiopian J Sci Sustainable Dev. (2020) 7:1–8. 10.20372/ejssdastu:v7.i1.2020.153

[38] Coppo E Marchese A. Antibacterial activity of polyphenols. Curr Pharm Biotechnol. (2014) 15:380–90. 10.2174/138920101504140825121142 25312620

[39] Cushnie TPT Lamb AJ. Antimicrobial activity of flavonoids. Int J Antimicrob Agents. (2005) 26:343–56. 10.1016/j.ijantimicag.2005.09.002 16323269 PMC7127073

[40] Sachla AJ Helmann JD. A bacterial checkpoint protein for ribosome assembly moonlights as an essential metabolite-proofreading enzyme. Nat Commun. (2019) 10:1526. 10.1038/s41467-019-09508-z 30948730 PMC6449344

[41] Davydova A Vorobjeva M Pyshnyi D Altman S Vlassov V Venyaminova A. Aptamers against pathogenic microorganisms. Crit Rev Microbiol. (2016) 42:847–65. 10.3109/1040841X.2015.1070115 26258445 PMC5022137

